# Dual Antibacterial Effect of In Situ Electrospun Curcumin Composite Nanofibers to Sterilize Drug-Resistant Bacteria

**DOI:** 10.1186/s11671-021-03513-2

**Published:** 2021-04-07

**Authors:** Chun-Li Liu, Jun Yang, Xiao-Han Bai, Zhi-Kai Cao, Chen Yang, Seeram Ramakrishna, Da-Peng Yang, Jun Zhang, Yun-Ze Long

**Affiliations:** 1 Collaborative Innovation Center for Nanomaterials and Devices, College of Physics, Qingdao University, Qingdao, 266071 China; 2 Center for Nanofibers and Nanotechnology, Department of Mechanical Engineering, National University of Singapore, Singapore, 117574 Singapore; 3 College of Chemical Engineering and Materials Science, Quanzhou Normal University, Quanzhou, 362000 China

**Keywords:** Electrospinning, In situ deposition, Drug-resistant bacteria, Curcumin, Naofibers

## Abstract

Bacterial infection especially caused by multidrug-resistant bacteria still endangers human life. Photodynamic therapy (PDT) can effectively kill bacteria, and nanofiber-based PDT can effectively reduce damage to normal tissues. However, current photosensitizers coated on the surfaces of fibers would release to the wound, causing some side effects. And nanofibers prepared by traditional method exhibit poor adhesion on the wound, which severely reduces the PDT effect due to its short-range effect. Herein, core–shell curcumin composite nanofibers are prepared by in situ electrospinning method via a self-made portable electrospinning device. The obtained composite nanofibers show superior adhesiveness on different biological surface than that of traditional preparation method. Upon 808-nm irradiation, these composite nanofibers effectively produced singlet oxygen (^1^O_2_) without curcumin falling off. After these composite nanofibers’ exposure to drug-resistant bacteria, they exhibit dual antibacterial behaviors and efficiently kill the drug-resistant bacteria. These dual antibacterial nanofiber membranes with excellent adhesiveness may benefit the application of wound infection as antibacterial dressing.

**Supplementary Information:**

The online version contains supplementary material available at 10.1186/s11671-021-03513-2.

## Background

Bacterial infection without timely treatment will cause septicemia, and sepsis thus seriously endangers life and health [1–3]. Although antibiotic can kill bacteria, using antibiotic in long term will lead to the development of drug-resistant bacteria, such as methicillin-resistant staphylococcus aureus (MRSA) [4–6]. MRSA, as a kind of multi-drug resistant bacteria, is one of the common bacteria that cause wound infection [7]. In this situation, it is necessary to find strategies to kill bacteria safely without developing resistance. It has already been proved that photodynamic therapy (PDT) is an effective method of sterilization [8–11]. However, most photosensitizers for PDT require ultraviolet light or short-wavelength excitation [12, 13]. Since the penetration depth of light in organism depends on the wavelength, the penetration depth of ultraviolet light and visible light is shallow, while the penetration depth of near-infrared (NIR) light is deep relatively. What’s worse, ultraviolet light and short-wavelength light will seriously burn human tissues. In order to achieve safe and antibacterial treatments in deep tissue, developing photosensitizers excited by NIR light is a demand and trend. Upconversion nanoparticles (UCNPs) can convert NIR light into short-wavelength light [14, 15]. Due to this property, photosensitizers can be designed to combine with upconversion to achieve NIR excitation. UCNPs are used as wavelength conversion station that converts NIR light to short wavelength to excite the photosensitizers and produce singlet oxygen (^1^O_2_) [16–19]. However, previous studies most on preparation of photosensitizers coated nanoparticle structure. Photosensitizers naked on the outermost layer of nanoparticles are easy to fall off [20, 21], and it also has some side effects on biological tissues because of direct contact, such as inhibiting tissue collagen growth [22, 23]. In fact, photosensitizers can achieve sterilization is due to its production of singlet oxygen, which means that there is no need for photosensitizers to direct contact with bacteria or biological tissues. Therefore, we can design a spacer to separate photosensitizers from biological tissues so that to avoid the possible side effects.

Electrospinning is a fast and efficient method to prepare nanofibers, including organic and inorganic nanofibers [24–28]. During the preparation process of nanofiber, nanoparticles are easy to combine with fibers to form composite nanofibers. There are mainly two methods to form composite nanofibers. One is doping particles inside the nanofibers [29], and the other is loading particles onto the surfaces of nanofibers [30, 31]. Considering the purpose of separating photosensitizers from biological tissues, incorporating photosensitizers into the nanofibers is more preferable compared to photosensitizers loaded on the fiber surfaces, which is easy to fall off. However, if nanofibers are hydrophobic that cannot infiltrate, the singlet oxygen are hard to produce and deliver to the fiber surfaces achieving antibacterial property [32]. But hydrophilic nanofibers are easy to dissolve when contaminated by interstitial fluid. Therefore, it is necessary to combine NIR photosensitizers with nanofibers and ensure the photodynamic nanofibers can effectively kill bacteria, especially drug-resistant bacteria.

In this study, curcumin is used as photosensitizers because of its wide sources from organism extracts. Core–shell nanostructure of UCNPs is used as wavelength transfer station, and it shows high conversion efficiency to produce ^1^O_2_. The UCNPs@Curcumin composite nanofibers are prepared by in situ electrospinning method via a self-made electrospinning device. The adhesion of the composite nanofibers obtained by this method on different biological surfaces is better than the traditional electrospinning preparation method. Upon 808-nm irradiation, these composite nanofibers can effectively produce ^1^O_2_ without curcumin falling off. After these composite nanofibers are contaminated with drug-resistant bacteria of MRSA, they will occur dual antibacterial behaviors that effectively kill the drug-resistant bacteria.

## Methods

### Materials

Thulium chloride, ytterbium chloride, neodymium chloride, and yttrium chloride were purchased from Sigma-Aldrich. Methanol, ethanol, cyclohexane, curcumin, dichloromethane, acetone, polyvinylpyrrolidone (PVP), polycaprolactone (PCL), and polyethyleneimine (PEI) were bought from Sinopharm Chemical Reagents. All materials were used without further purification.

### Synthesis of Core–Shell NaYF_4_:Yb/Tm@NaYF_4_:Nd@Curcumin

Upconversion nanoparticles (UCNPs) of NaYF_4_:Yb/Tm@NaYF_4_:Nd were synthesized using co-precipitate methods [33, 34]. Afterward, 200 mg of as-prepared UCNPs, 90 mg of PEI, and 180 mg of curcumin were added and dissolved in methylene dichloride. The reactants were stirred uniformly for 20 h at room temperature, and obtained products were purified by centrifugation and washed twice by ethanol.

### Preparation of Curcumin Composite Nanofibers Via In Situ Electrospinning

One gram of PCL, 0.16 g of PVP, and 0.1 g of NaYF_4_:Yb/Tm@NaYF_4_:Nd@Curcumin were added into 5 mL of acetone. After 12 h of stirring, a homogeneous precursor solution was obtained for electrospinning. Taking 3 mL of the precursor solution in 5-mL syringe, a self-made handheld electrospinning equipment was used for electrospinning, which consists of a 0.4-mm metal needle in diameter, two alkaline batteries, and a high-voltage converter that can convert 3 V of battery to 10 kV for electrospinning. The electrospinning distance between collector and electrospinning needle was about 10 cm.

### ***Detection of ***^***1***^***O***_***2***_*** Formation***

Singlet oxygen sensor green (SOSG) was utilized to detect the ^1^O_2_ formation. An 9 × 9 mm square of as-prepared nanocomposite fiber membrane with different concentration of UCNPs@Curcumin was added in a quartz cuvette, and then 3 mL of methanol containing 25 μM of SOSG was added. Afterward, the cuvette was irradiated under the 808-nm laser with different irradiation time. The fluorescence spectrophotometer with 504 nm of excitation wavelength was used to measure the fluorescence intensity of this solution, which reflects the singlet oxygen level.

### Antibacterial Assay

Drug-resistant bacteria of MRSA and Escherichia coli were used to evaluate the antibacterial ability. Briefly, bacterial strains were cultivated in the tryptic soy broth medium. The culture media containing bacterial strains were incubated at 37 °C for 15 h. After culturing, the concentration of bacterial strain was 1 × 10^6^ CFU/mL. In total, 100 μL of bacterial solution was placed in each well of 96-well plate on a sterile ultra-clean table. Then, a piece of circular fiber membrane with 6 mm diameter was added to each well of the 96-well plate. After 20 min of 808-nm laser irradiation, the bacterial solution in the plate was diluted 10 times with sterile water. A 10 μL of diluent was placed in a nutritional agar plate to obtain an evenly coated agar plate. The treated agar plate was cultured in a constant temperature bacterial incubator at 37 °C for 18 h and then took photographs. In the control group, the steps were the same as above, except that 808-nm laser irradiation was not used. Each group was repeated with 5 plates.

### Characterization

TEM and SEM images were taken from JEM-2010 and SU-1510 electron microscopes. Fluorescence spectrum was measured on Edinburgh FLS1000 fluorescence spectrophotometer. The absorption spectrum was recorded on Shimadzu UV2550 spectrometer. Fourier transform infrared spectroscopy was taken on Nicolet iS50 spectrometer. The zeta potential was measured with WJL-608 analyzer. The hydrophilicity with sessile drop method was tested by PT-602Atest equipment.

## Results and Discussion

### Characterization of Nanoparticles and Composite Nanofibers

**Fig. 1:**
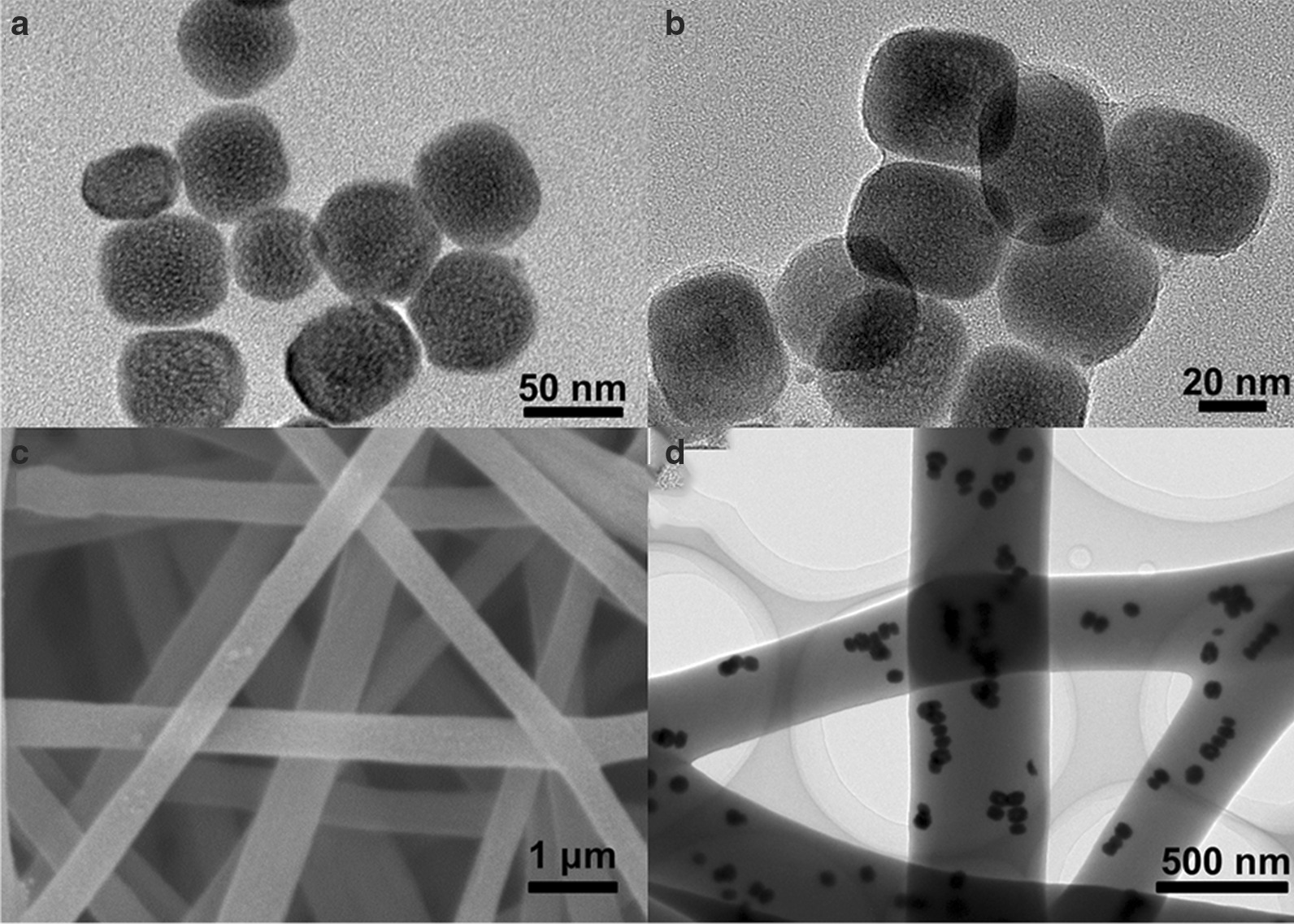
TEM images of **a** NaYF_4_:Yb/Tm@NaYF_4_:Nd nanoparticles (UCNPs) and **b** core–shell structured UCNPs@Curcumin nanoparticles. **c** SEM image of curcumin composite nanofibers, **d** TEM image of curcumin composite nanofibers

Figure 1a shows the TEM image of NaYF_4_:Yb/Tm@NaYF_4_:Nd nanoparticles (UCNPs). It demonstrates a uniform size distribution of UCNPs with an average diameter of about 45 nm. The zeta potential of these nanoparticles was further tested to be + 19 mV (Addition file 1: Fig. S1). After the UCNPs were coated with curcumin, Fig. 1b shows a core–shell structure and the curcumin shell thickness is about 5 nm. Afterward, these core–shell curcumin nanoparticles were embedded into PCL/PVP fibers. Figure 1c shows the SEM image of these composite nanofibers prepared by a self-designed handheld electrospinning device. The diameter of the continuous and non-fracture nanofibers prepared by this device is about 400 nm, and the fiber uniformity is similar to that of traditional electrospinning devices (Addition file 1: Fig. S2). It should be noted that this portable electrospinning device can be operated by two dry batteries of 1.5 V (Addition file 1: Fig. S3), which gets rid of the limitation of using city power supply. Combined with its other advantages of light weight (160 g in weight) and small size, it will benefit outdoor usage. Figure 1d shows the TEM image of these composite nanofibers, indicating that the nanoparticles have good dispersibility in the nanofibers.

**Fig. 2:**
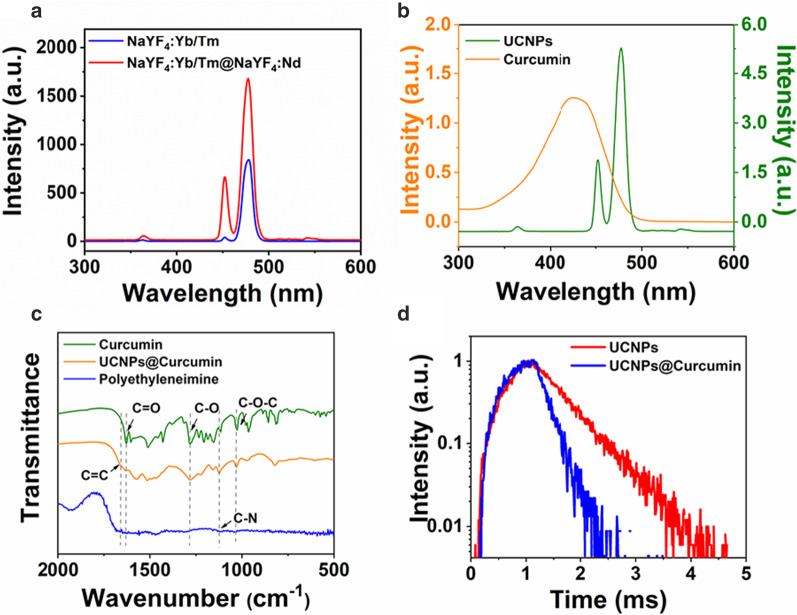
**a** Fluorescence spectrum of core–shell NaYF_4_:Yb/Tm@NaYF_4_:Nd excited by 808 nm, **b** fluorescence spectrum of UCNPs and UV–vis absorption spectrum of curcumin, **c** FTIR spectra of UCNPs@Curcumin, curcumin and PEI, **d** time-resolved fluorescence spectra of UCNPs and UCNPs@Curcumin

The reason for coating NaYF_4_:Nd shell on the NaYF_4_:Yb/Tm core was that it can enhance photoluminescence (Fig. 2a). Because the fluorescence spectra of UCNPs overlapped well with the UV–Vis absorption spectra of curcumin (Fig. 2b), it is meant that stronger photoluminescence of UCNPs can transfer more energy to curcumin, which was conducive to the excitation of photosensitizers. Furthermore, considering that NIR light with a wavelength of 808 nm penetrates more deeply into living tissue than NIR light with a wavelength of 980 nm, the introduction of this NaYF_4_:Nd shell can modulate the excitation wavelength from 980 to 808 nm (Additional file 1: Fig. S4), thereby reducing undesirable burns on normal tissue. FTIR measurement was further measured. As can be seen from Fig. 2c, stretch vibrations of C=O at 1628 cm^−1^, C–O at 1282 cm^−1^, and C–O–C at 1028 cm^−1^ occur in the nanocomposite particles (orange line), which origin from curcumin (green line). Meanwhile, there is a stretching vibration of C–N at 1125 cm^−1^, which comes from the PEI (blue line). Their molecular structure diagrams are illustrated in appendix (Addition file 1: Fig. S5). Moreover, there is a weak C=C at approximately 1660 cm^−1^, which corresponds to the oleic acid at the same time of synthesis of UCNPs. It can demonstrate the components of UCNPs@Curcumin composite nanofibers.

Figure 2d exhibits fluorescence attenuation curves of UCNPs before and after coating curcumin. It shows that the fluorescence lifetime of UCNPs decreased from 700 to 390 μs after coating with curcumin shells. On the basis of *γ* = 1 − *τ*_2_/*τ*_1_, where *τ*_2_ and *τ*_1_ are the lifetime of UCNPs before and after the envelope of curcumin, *γ* is the energy transfer efficiency [35]. Thus, *γ* was calculated to be 44.3%. Such high energy transfer efficiency was obtained, which on the first aspect was due to the good overlaps between absorption spectra of curcumin and photoluminescence spectra of UCNPs (Fig. 2b), so that non-radiative energy transfer can occur between them. The second aspect was that UCNPs had a NaYF_4_:Nd shell that enhances the fluorescence intensity, thus increasing their spectral overlap integral area. The third aspect was that the distance between curcumin and UCNPs was the coating thickness (< 5 nm), and this small distance was conducive to the generation of highly efficient fluorescence resonance energy transfer (FRET). The FRET method can obtain as high as 44.3% energy transfer efficiency, which can also benefit the following efficient production of ^1^O_2_.

### Producing ^1^O_2_ from Composite Nanofibers

**Fig. 3:**
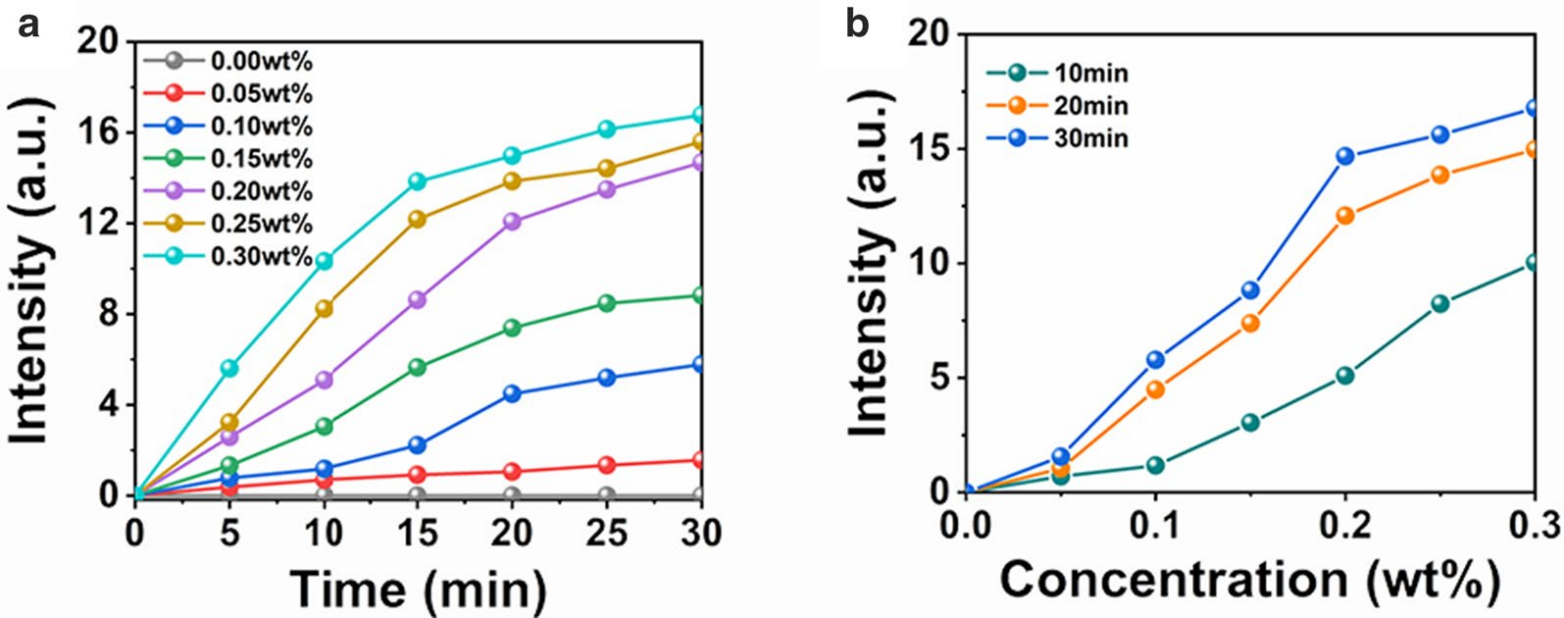
Singlet oxygen production of composite nanofiber membrane doped with UCNPs@Curcumin exposed to 808-nm light at different **a** concentration and **b** irradiation time

In order to evaluate the ability of nanocomposite fibers to produce ^1^O_2_, the SOSG method was used. First, we took nanocomposite fibers with a fixed doping concentration and observed the generation of ^1^O_2_ under different irradiation time. As shown in Fig. 3a, for a fixed concentration such as 0.20 wt%, irradiation time is one of the factors affecting the generation of ^1^O_2_. The longer the irradiation time, the more ^1^O_2_ was produced. However, it also shows that although the concentration of ^1^O_2_ gradually increases with the increase in irradiation time, the rising rate slows down gradually and almost remains constant after 20 min, which is manifested by a dense curve interval. This phenomenon may be due to the fast local oxygen consuming by producing ^1^O_2_ with sustained NIR light radiations, resulting in a relatively low oxygen level in local area, and thus decreasing the rising rate of producing ^1^O_2_.

To observe the influence of doping concentration on producing ^1^O_2_, Fig. 3b is further depicted. As shown in Fig. 3b, for a fixed irradiation time such as 20 min, with increasing the doping concentration, more ^1^O_2_ was produced. However, the rising rate of ^1^O_2_ slowed down when the concentration was larger than 0.20 wt%. These experimental results suggest that there is no need to infinitely increase the irradiation time and doping concentration to produce more ^1^O_2_. The optimal choice is 0.20 wt% with 20 min and thus in the following experiments will take this concentration and irradiation time.

### Wettability and Adhesivity of In Situ Electrospun Nanofiber Membrane

**Fig. 4:**
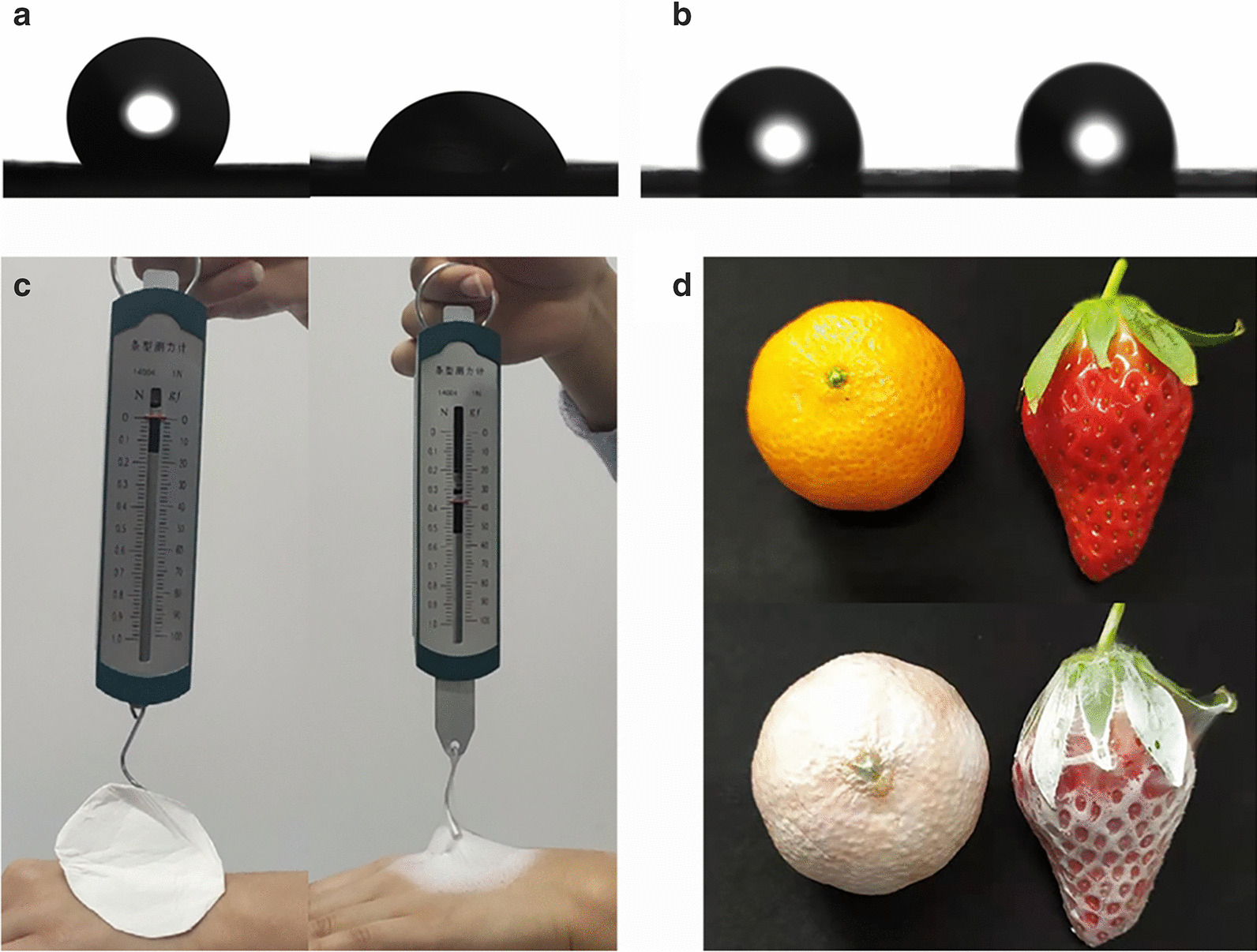
Water contact angle measurement of composite nanofiber membrane with matrix of **a** PCL/PVP and **b** PCL, **c** traditional electrospun nanofiber membrane and in situ deposition electrospun nanofiber membrane, **d** in situ deposition electrospun on different object surface

Considering producing ^1^O_2_ is a process that requires UCNPs@Curcumin nanoparticles in fibers to interact with oxygen in body fluid, thus the contact angle of this fiber membrane was further tested. Figure 4a shows a drop of water dropped onto the surface of this composite nanofiber membrane and its wettability after 20 s. Compared with pure PCL nanofiber membrane (Fig. 4b), the composite nanofiber membrane has better wettability. Interestingly, after soaking composite nanofiber membrane in phosphate buffer solution (PBS), there was no UCNPs@Curcumin detected in PBS by absorption spectrometer, which is meant that no curcumin was shed from the fibers. The reason may be that curcumin was coated onto the UCNPs, so the size of UCNPs@Curcumin (~ 50 nm) was too large to penetrate the fiber. Compared with methods of photosensitizers coated on the particles or fibers, increasing the size of curcumin first and then doping it into the wetting fibers can effectively avoid the shedding of photosensitizers and enhance the generation and diffusion of ^1^O_2_. In addition, considering that the short-range effect of PDT and the poor adhesion of the fiber membrane prepared by the traditional electrospinning method to the wound surface (Fig. 4c; Additional file 1: Fig. S6), the photodynamic effect would be affected due to the interval between the fiber membrane and the surface. Fortunately, these curcumin composite nanofibers could be prepared by in situ electrospining method with good morphology (Fig. 1c) and also exhibited good adhesivity on different object surfaces (Fig. 4d). It means that the in situ electrospinning deposition method to prepare the photodynamic fiber membrane is more preferable than the traditional spinning method in which the fiber membrane is collected on the foil and then pressed on the wound surface.

### Dual Antibacterial Effect of Curcumin Composite Nanofibers

**Fig. 5:**
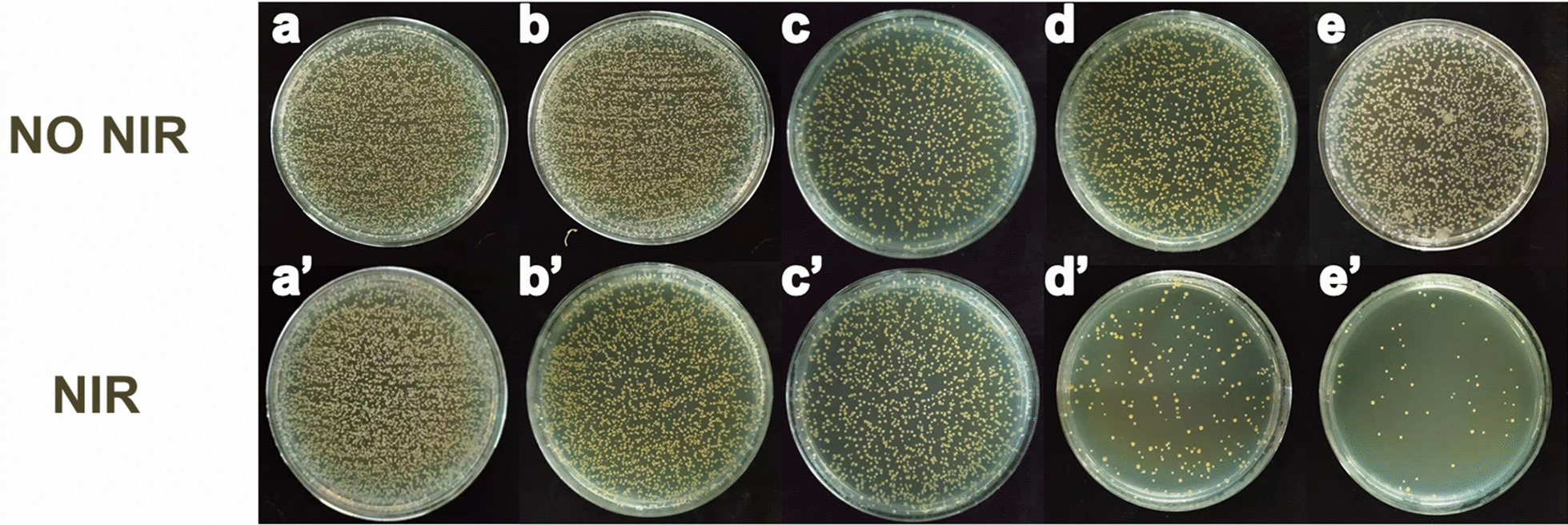
Antibacterial performance of nanofiber doped with different samples against MRSA **a**–**e** without and **a′**–**e′** with 808-nm light exposure: **a**, **a′** control group, **b**, **b′** UCNPs group, **c**, **c′** curcumin group, **d**, **d′** UCNPs@Curcumin with low-dose group, and **e**, **e′** high-dose group

The nanocomposite fibers prepared by the device have been proved to be non-toxic by MTT assay (Addition file 1: Fig. S7). Futher, in order to prove the fibers have good antibacterial properties, the count method was used to evaluate the antibacterial properties of composite nanofibers. As shown in Fig. 5, whether or not 808-nm light is irradiated on pure fibers, there is no antibacterial property (Fig. 5a, b). These results show that 808-nm light itself has no bactericidal effect. When the fibers are doped with UCNPs, the bacteria do not decrease, which confirms that UCNPs have no bactericidal effect (Fig. 5a′, b′). Interestingly, when the fibers are doped with curcumin, the number of bacteria decreases to a certain extent, which proves that curcumin itself shows certain antibacterial activity (Fig. 5c, c′). Furthermore, an obvious bactericidal result occurred in fibers doped with UCNPs@Curcumin under NIR light irradiation (Fig. 5d′, e′). Combined with the results of Fig. 3, these bactericidal results indicate that the ^1^O_2_ produced from UCNPs@Curcumin under 808-nm irradiation could effectively kill bacteria. On the other hand, the antibacterial activity of curcumin was the same in the presence and absence of 808-nm irradiation, due to the fact that the absorbance of curcumin was in the visible light range (Fig. 2b), so 808-nm light was not effective. This was also the reason why curcumin was designed to coat the surface of UCNPs. In addition, Fig. 5d, e shows fibers doped with UCNPs@Curcumin at 0.15 wt% and 0.20 wt%, respectively. By comparison, it is found that the 0.20 wt% group exhibited better bactericidal properties at 20 min of light irradiation, and the antibacterial effect reached 95%. This is because the ^1^O_2_ produced by the photosensitizer curcumin in the photodynamic effect can kill drug-resistant bacteria. This result is also consistent with the ^1^O_2_ result in Fig. 3. These data further indicate that fibers doped with UCNPs@Curcumin can kill MRSA due to its dual antibacterial activity, namely fibers doped with UCNPs@Curcumin and PDT, and PDT has better antibacterial effect than fibers doped with UCNPs@Curcumin. In addition, we also conducted experiments with Escherichia coli, which also confirmed that in situ electrospun curcumin composite nanofibers have dual antibacterial effects on drug-resistant bacteria (Addition file 1: Fig. S8). And the anti-inflammatory effect of the nanofibers was further verified by H&E staining of MRSA (Addition file 1: Fig. S9). After different treatment of wound infection, a large number of neutrophils were collected in the without nanocomposite fiber group, which were purple and blue cell clusters due to tissue injury and suppurative infection. However, a small amount of granulation tissue and red blood cells appeared in the nanofiber group, which indirectly reflected the antibacterial properties of nanocomposite fibers. It has a blocking effect on inflammation of wound infection.

## Conclusions

In summary, core–shell curcumin composite nanofibers are prepared by in situ electrospinning method via a self-made portable electrospinning device. The obtained composite nanofibers show superior adhesiveness on different biological surfaces than that of traditional preparation method. The method, firstly increasing the size of curcumin followed by doping it into the wettable fiber, can effectively avoid the shedding of photosensitizers, thus enhancing the producing of ^1^O_2_ and its diffusion, which may provide inspiration for designing other photodynamic nanomaterials. After these composite nanofibers contaminated with drug-resistant bacteria, they exhibit dual antibacterial behaviors and efficiently kill the drug-resistant bacteria. These dual antibacterial nanofiber membranes have excellent adhesion and can be used as antibacterial dressings in combination with hemostasis, thus enabling outdoor hemostasis.

## Supplementary Information

**Additional file 1: Figure S1.** SEM image of nanofibers obtained by traditional electrospinning device. **Figure S2.** A light, small in size and portable electrospinning device powered by battery is developed for outdoor use (160 g in total weight), and its structure diagram. **Figure S3.** Schematic illustration of energy transfer and pathway from NaYF_4_:Yb/Tm@NaYF_4_:Nd shell to NaYF_4_:Yb/Tm core under 808-nm laser excitation. **Figure S4.** Structural formula of curcumin, oleic acid, and PEI. **Figure S5.** Tensile test of curcumin composite nanofibers prepared by different method.

## Data Availability

The datasets generated during and/or analyzed during the current study are available from the corresponding authors on reasonable request.

## Abbreviations

PDT: Photodynamic therapy
^1^O_2_: Singlet oxygen
MRSA: Methicillin-resistant staphylococcus aureus
NIR: Near-infrared
UCNPs: Upconversion nanoparticles
PVP: Polyvinylpyrrolidone
PCL: Polycaprolactone
PEI: Polyethyleneimine
SOSG: Singlet oxygen sensor green
FRET: Fluorescence resonance energy transfer
PBS: Phosphate buffer solution
