# Supplementary material for: Dual Antibacterial Effect of In Situ Electrospun Curcumin Composite Nanofibers to Sterilize Drug-Resistant Bacteria
Source: Nanoscale Res Lett. 2021 Apr 7;16:54. doi: 10.1186/s11671-021-03513-2 (PMC8026794; doi:10.1186/s11671-021-03513-2)
Supplement: Supplementary file 1 — Additional file 1: Figure S1. SEM image of nanofibers obtained by traditional electrospinning device. Figure S2. A light, small in size and portable electrospinning device powered by battery is developed for outdoor use (160 g in total weight), and its structure diagram. Figure S3. Schematic illustration of energy transfer and pathway from NaYF4:Yb/Tm@NaYF4:Nd shell to NaYF4:Yb/Tm core under 808-nm laser excitation. Figure S4. Structural formula of curcumin, oleic acid, and PEI. Figure S5. Tensile test of curcumin composite nanofibers prepared by different method. [file 11671_2021_3513_MOESM1_ESM.docx]

Supporting Information

**Dual Antibacterial Effect of *In-situ* Electrospun Curcumin Composite Nanofibers to Sterilize Drug-Resistant Bacteria**

Chun-Li Liu^1,†^, Jun Yang^1,†^, Xiao-Han Bai^1^, Zhi-Kai Cao^1^, Chen Yang^1^, Seeram Ramakrishna^2^, Da-Peng Yang^3^, Jun Zhang ^1,^* and Yun-Ze Long^1,^*

^1^ *Collaborative Innovation Center for Nanomaterials & Devices, College of Physics, Qingdao University, Qingdao 266071, China*

^2^ *Center for Nanofibers & Nanotechnology, Department of Mechanical Engineering, National University of Singapore, Singapore 117574, Singapore*

^3^ *College of Chemical Engineering and Materials Science, Quanzhou Normal University, Quanzhou 362000, China*

______________________

^*^Corresponding author.

Fax: +86-532-8595 5977. Tel: +86-139 5329 0681.

E-mail: iamjunzhang@163.com or yunze.long@qdu.edu.cn

^†^These two authors contributed equally to this work.


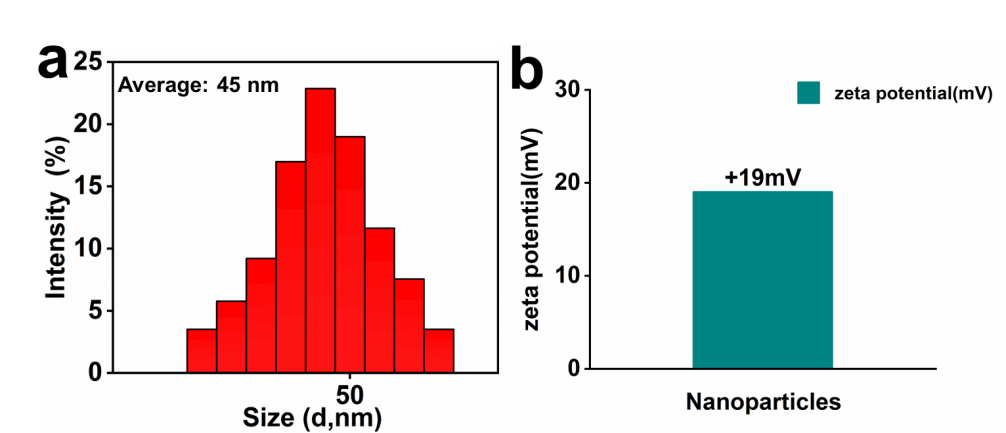


**Figure S1. a** Particle size distributions and **b** zeta potential of the nanoparticles.


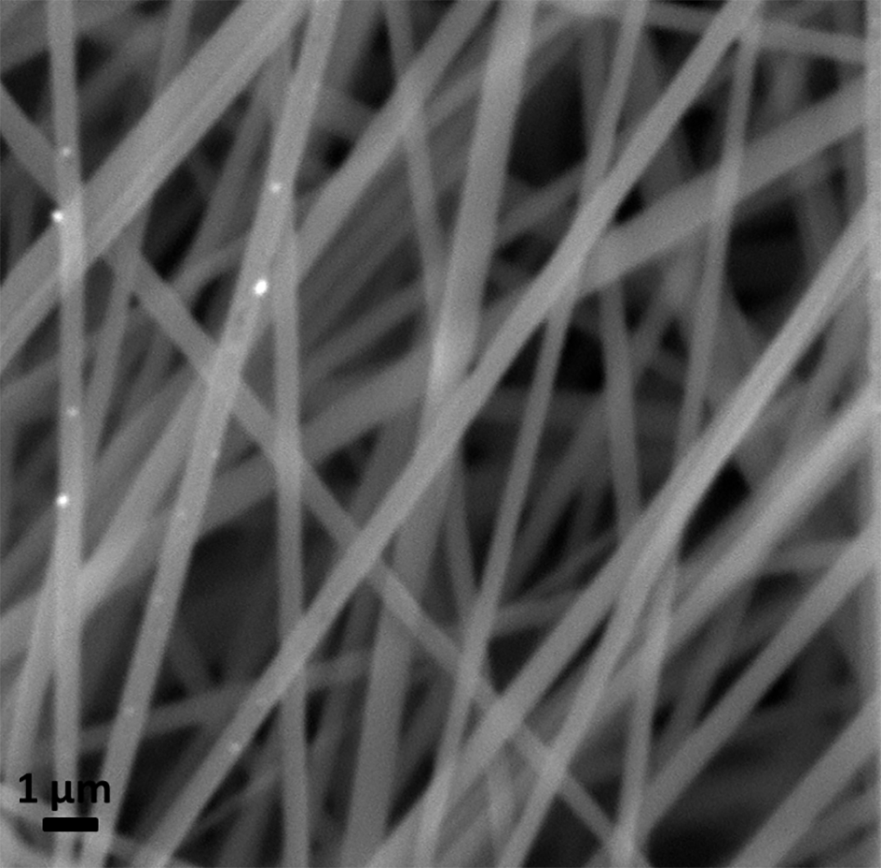


**Figure S2.** SEM image of nanofibers obtained by traditional electrospinning device.


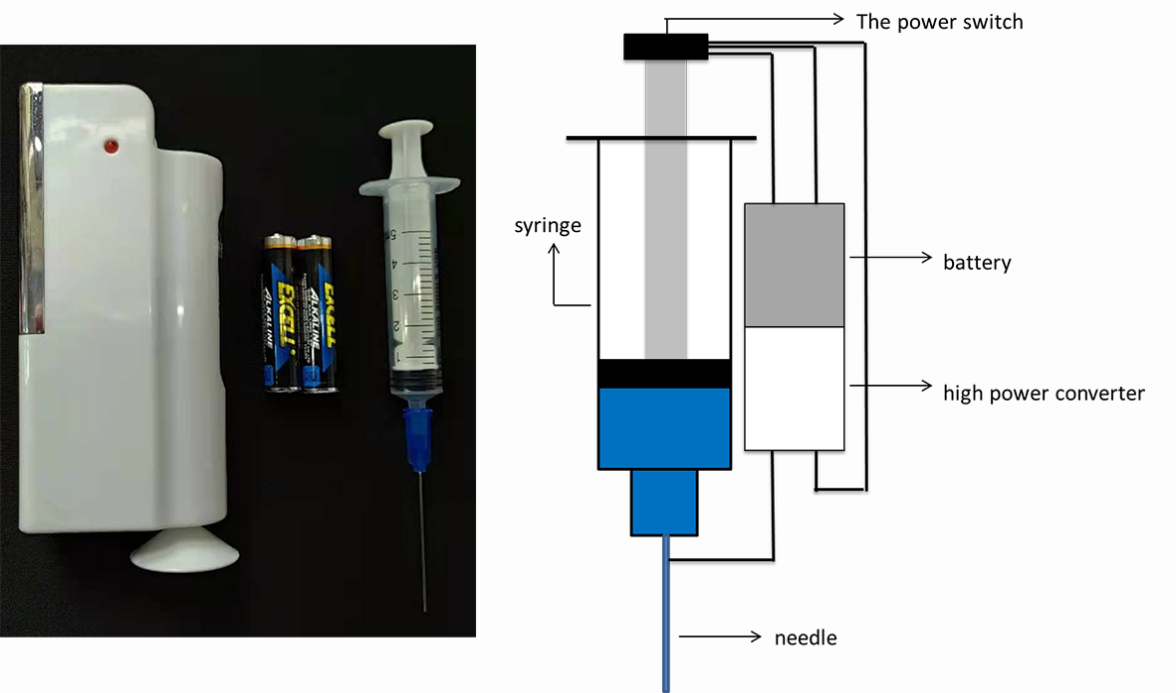


**Figure S3.** A light, small in size and portable electrospinning device powered by battery is developed for outdoor use (160 g in total weight), and its structure diagram.


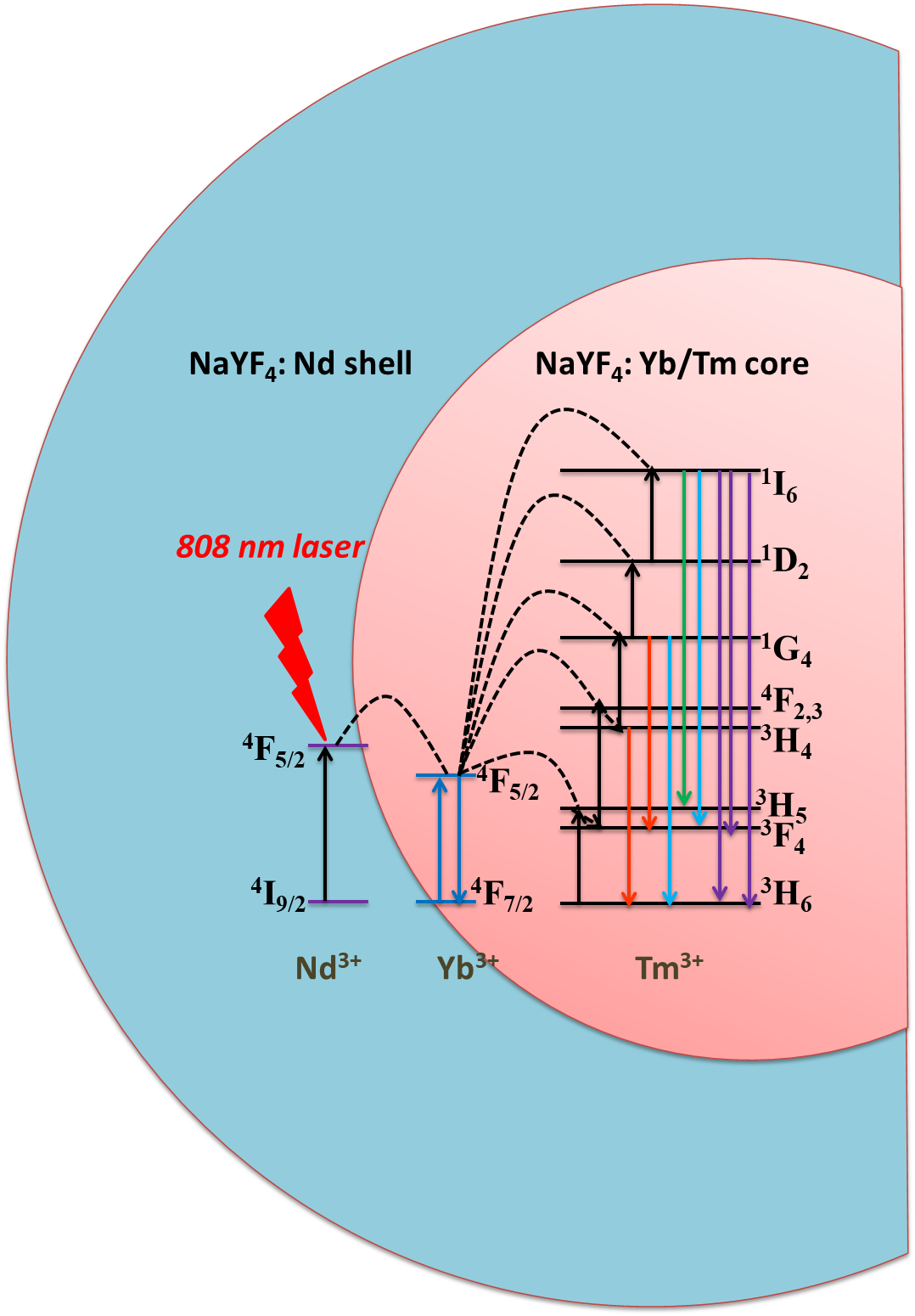


**Figure S4.** Schematic illustration of energy transfer and pathway from NaYF_4_:Yb/Tm@NaYF_4_:Nd shell to NaYF_4_:Yb/Tm core under 808 nm laser excitation.


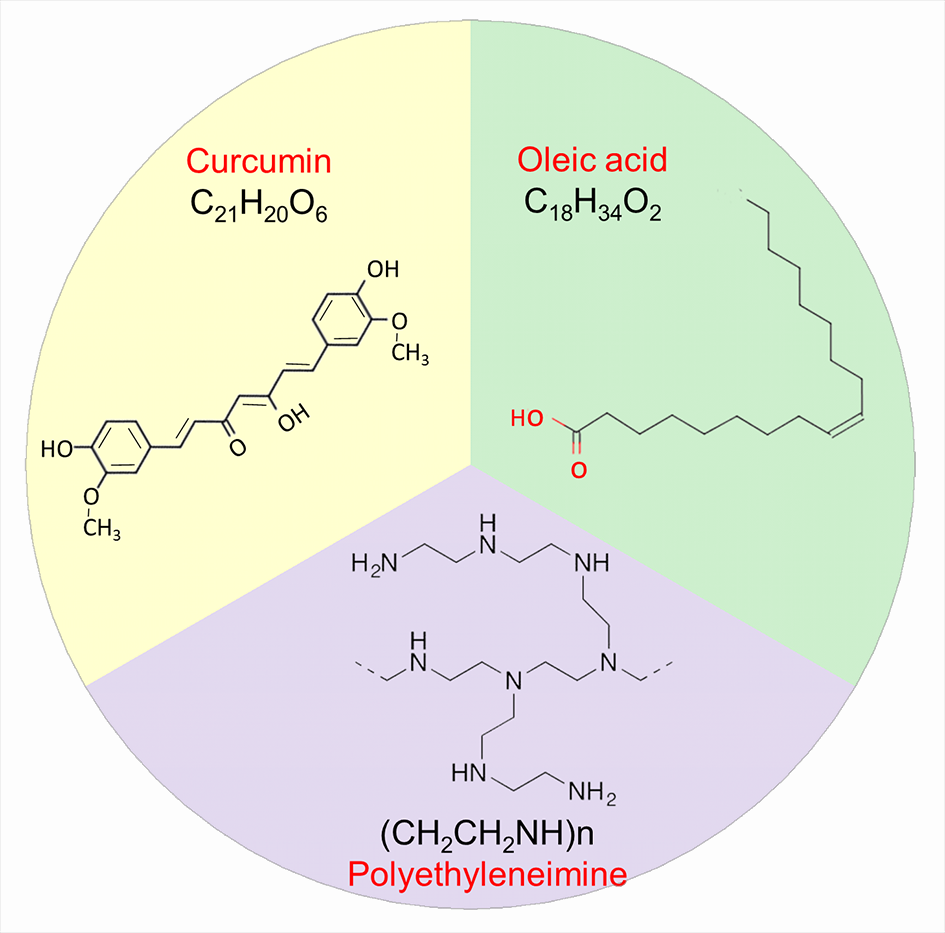


**Figure S5.** Structural formula of curcumin, oleic acid, and PEI.


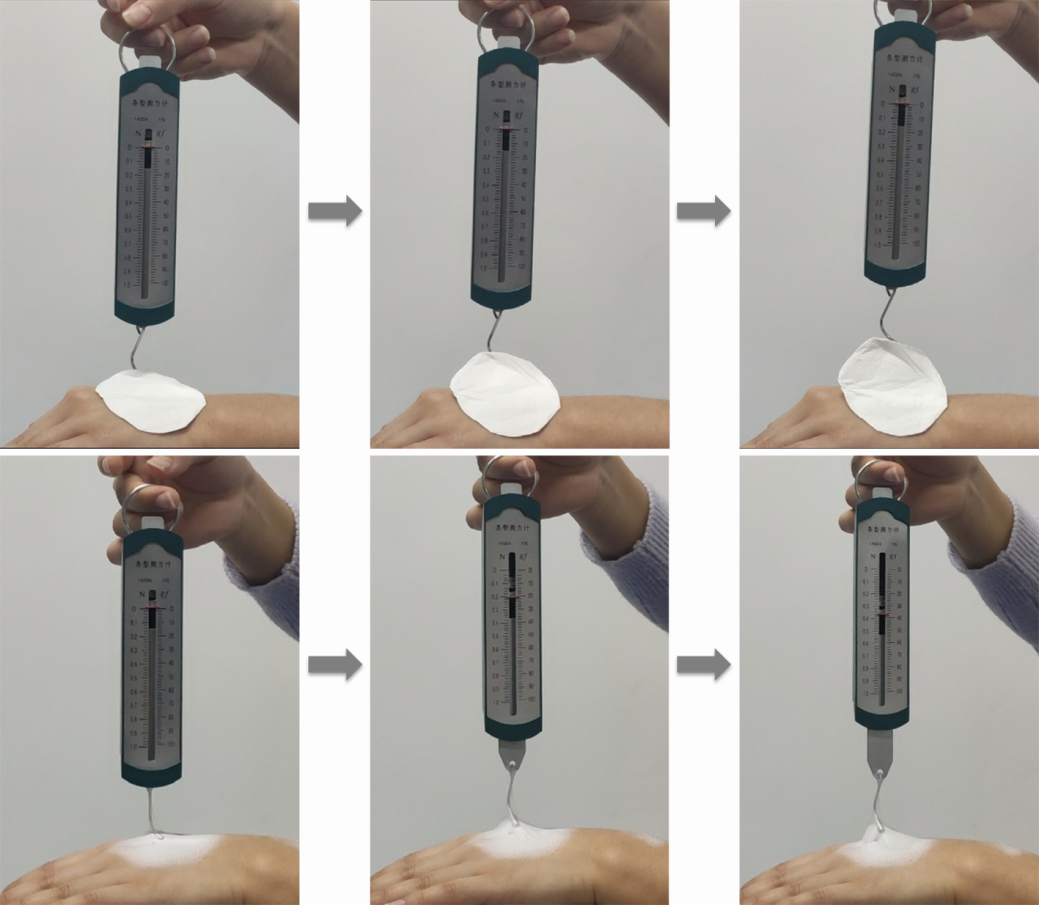


**Figure S6.** Tensile test of curcumin composite nanofibers prepared by the traditional electrospinning method that fiber membranes are in vitro prepared followed by pressing onto the skin, and the in situ electrospinning method.


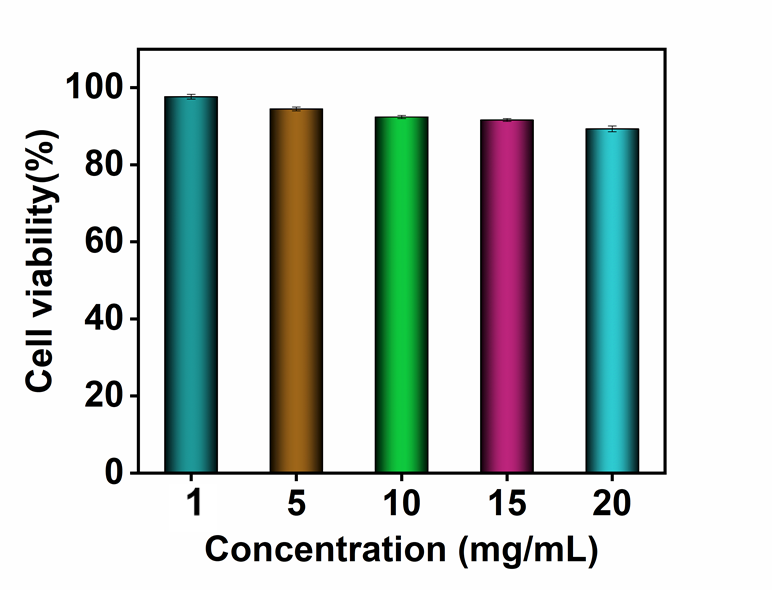


**Figure S7.** The biocompatibility of nanocomposite fiber membrane analyzed using MTT assay. The L929 fibroblast cells incubated with fiber membranes for 36 h.


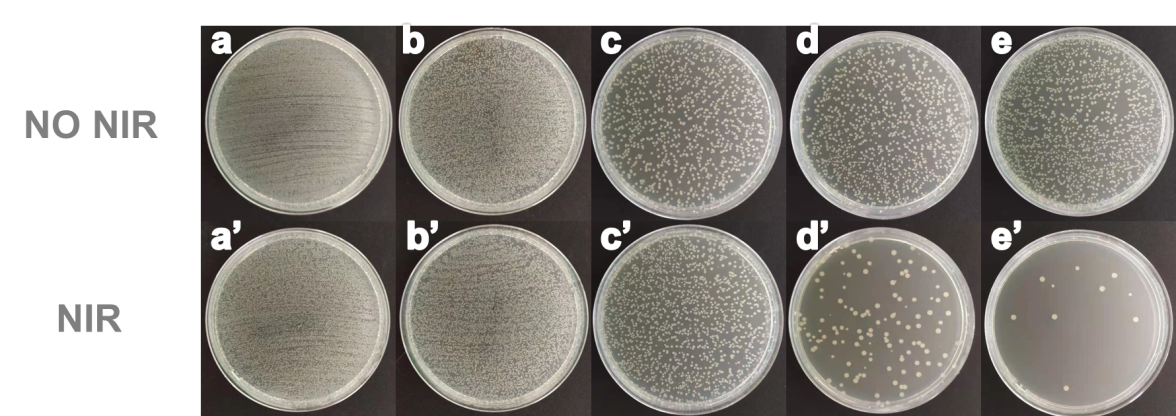


**Figure S8.** Antibacterial performance of nanofibers doped with different samples against Escherichia coli **a**-**e** without and **a’**-**e’** with 808 nm light exposure: **a**, **a’** control group, **b**, **b’** UCNPs group, **c**, **c’** curcumin group, **d**, **d’** UCNPs@Curcumin with low dose group, and **e**, **e’** high dose group.


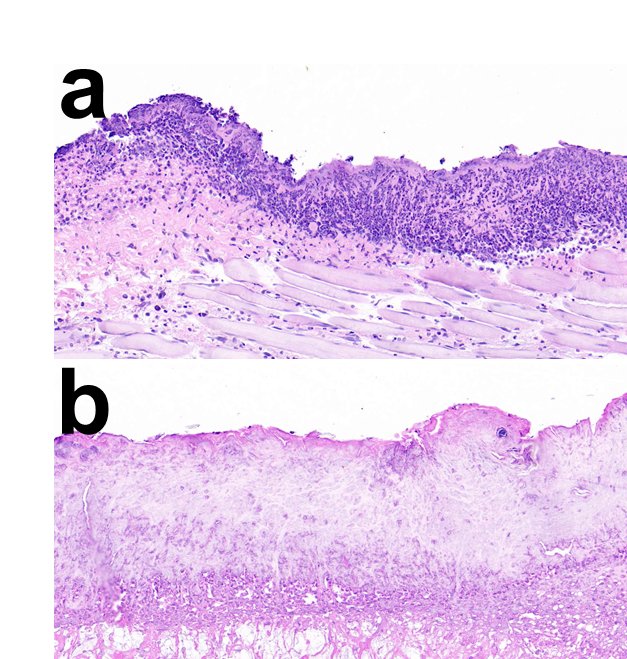


**Figure S9.** H&E staining of MRSA infected wound tissue sections treated **a** without nanocomposite fibers and **b** with nanocomposite fibers.
